# Proficiency test of BRAF immunohistochemistry as a surrogate marker of p.V600E mutation: Assessment of staining and interpretation quality in Taiwan

**DOI:** 10.1007/s00428-025-04192-5

**Published:** 2025-07-21

**Authors:** Yun-An Chen, Jyie-Yu Lai, Chih-Yi Hsu, Huang-Chun Lien, Jen-Fan Hang

**Affiliations:** 1 Department of Pathology, China Medical University Hospital, Taichung, Taiwan; 2 Taiwan Society of Pathology, Taipei, Taiwan; 3 Department of Pathology and Laboratory Medicine, Taipei Veterans General Hospital, Taipei, Taiwan; 4 Department of Pathology, School of Medicine, National Yang Ming Chiao Tung University, Taipei, Taiwan; 5 College of Nursing, National Taipei University of Nursing and Health Sciences, Taipei, Taiwan; 6 Department of Pathology, National Taiwan University Hospital, Taipei, Taiwan; 7 Graduate Institute of Pathology, National Taiwan University, Taipei, Taiwan; 8 Institute of Clinical Medicine, National Yang Ming Chiao Tung University, Taipei, Taiwan

**Keywords:** BRAF, p.V600E, VE1, Immunohistochemistry (IHC), Proficiency testing

## Abstract

BRAF immunohistochemistry (IHC) serves as a surrogate for *BRAF* p.V600E but shows variable performance across tumor types and institutions. This study evaluated BRAF IHC staining quality and interpretation in general pathology laboratories through a nationwide proficiency test (PT) in Taiwan, focusing on the most commonly encountered thyroid neoplasm and colorectal cancer. This PT was organized by the Taiwan Society of Pathology using a tissue microarray, containing six tumor cores with confirmed *BRAF* mutation status and one positive control. Participating laboratories performed BRAF IHC staining and interpretation independently, with results centrally reviewed for concordance, accuracy, and staining quality. Twenty-six pathology laboratories participated. Two laboratories failed the initial control check. Among the remaining 24, 17 (70.83%) demonstrated optimal staining, while 5 (20.83%) showed over-staining and 2 (8.33%) under-staining. No significant associations were found between staining quality and antibody clones, platforms, dilution folds, or assay types. Interpretation was highly concordant (100% agreement and accuracy) for tissues with 3 + or negative staining. However, discrepancies arose in tissues with 2 + intensity (50% positive, 41.67% negative, 8.33% equivocal) and 1 + intensity (83.33% disagreement with test results). The overall accuracy was 79.2%, with sensitivity at 58.3% and specificity at 100%. Under-calling was frequent in cases with 1 + staining (33 cores across 23 laboratories) and 2 + staining (3 cores across 3 laboratories). Our study highlights the importance of optimizing staining quality and reinforcing education on interpretation criteria. To minimize false-negative results, we recommend molecular confirmation for all cases exhibiting diffusely weak staining.

## Introduction

The *BRAF* gene encodes a serine/threonine kinase in the MAPK signaling pathway and activating mutations promote oncogenic proliferation in various cancers. Among solid tumors, *BRAF* mutations are most frequently observed in thyroid cancer (43.3%), melanoma (29.4%), and colorectal cancer (11.4%). Class 1 mutations, including p.V600E, p.V600K, and p.V600R, account for the majority of *BRAF* mutations, with p.V600E being the most common [1]. The *BRAF* p.V600E mutation has important prognostic and therapeutic implications. In 2022, the U.S. Food and Drug Administration approved combined dabrafenib and trametinib as tumor-agnostic treatment for all solid tumors harboring this mutation, except colorectal cancer [2]. In colorectal cancer (CRC), this mutation is linked to poor prognosis, resistance to anti-EGFR therapy [3] and help rule out Lynch syndrome in mismatch repair-deficient cases [4].

Detection methods for *BRAF* p.V600E include Sanger sequencing, qPCR, next-generation sequencing (NGS), and immunohistochemistry (IHC). While NGS and qPCR are highly sensitive, they can be costly or less accessible. IHC is widely adopted in routine pathology due to its cost-effectiveness, rapid turnaround, and ability to provide spatial information, especially in low-tumor-content samples [5–7]. Since Capper et al. developed the mutation-specific VE1 antibody in 2011[8], IHC has been evaluated as a surrogate for molecular testing, though results vary by tumor type and technical factors, with inter-study inconsistencies and occasional discordance with sequencing [9–11].

Melanoma, CRC and papillary thyroid carcinoma (PTC) are among the most frequently studied cancer types for BRAF IHC [5]. Studies have shown that BRAF IHC generally performs reliably in melanoma and PTC, while findings in CRC have been inconsistent, with significant variability across studies [12–18]. Previous studies have linked staining quality to technical factors [16, 17, 19, 20] and evaluated different interpretive criteria [16, 21]. However, most of these investigations were conducted at single institutions, and the available meta-analyses are limited in their ability to standardize staining performance or quantify inter-laboratory concordance due to variability in specimen types and staining protocols.

In Taiwan, thyroid cancer and CRC are among the top ten malignancies [22] and are most commonly encountered in BRAF IHC interpretation, while cutaneous melanoma is a rare disease. We therefore conducted a nationwide proficiency test (PT) in Taiwan, focusing on BRAF IHC for these two tumor types. Our goals were to evaluate staining quality, interpretation consistency, and inter-laboratory concordance across multiple pathology laboratories.

## Materials and methods

The study complied with local ethical regulations, and Institutional Review Board approval was exempted. A tissue microarray (TMA) was constructed with tissue cores of 2.0-mm diameter formalin-fixed paraffin-embedded tumor tissue. The TMA consisted of six cores of tumor tissues with molecularly confirmed *BRAF* mutation status, and one core of respiratory mucosa was included as a positive control [23]. The overall TMA design was adapted from the framework used in Nordic Immunohistochemical Quality Control (NordiQC) with similar core number [24]. However, in light of prior studies indicating inconsistent BRAF IHC performance in CRC, four of the six cores were selected from CRC cases with a range of staining intensities (1 + to 2 +) to better reflect diagnostic challenges in this tumor type. The original assessment of the immunohistochemical staining at the reference lab was performed using the VE1 in vitro diagnostic (IVD) monoclonal antibody (Ventana Medical Systems, Tucson, AZ, USA) at a ready-to-use concentration, on the Ventana BenchMark ULTRA automated staining platform. The details of each test sample are listed in Table 1.

**Table 1:** Summary of the testing cores

	Tissue	Diagnosis	Sequencing Result	Consensus Staining Result
Core 1	Thyroid	NIFTP	*BRAF* WT (NRAS p.Q61R)	Negative (0)
Core 2	Thyroid	Papillary carcinoma	*BRAF* p.V600E	Positive (3 +)
Core 3	Colon	Adenocarcinoma	*BRAF* WT (NRAS p.G13R)	Negative (0)
Core 4	Colon	Adenocarcinoma	*BRAF* p.V600E	Positive (2 +)
Core 5	Colon	Adenocarcinoma	*BRAF* p.V600E	Positive (1 +)
Core 6	Colon	Adenocarcinoma	*BRAF* WT (KRAS p.G12D)	Negative (0)

*NIFTP* noninvasive follicular thyroid neoplasm with papillary-like nuclear features; *WT* wild type

The 2024 BRAF IHC stain PT activity was organized by the Proficiency Testing Committee of the Taiwan Society of Pathology and distributed to registered institutions. Each participating laboratory received one unstained, 3 μm-thick slides of the TMA. Participants performed BRAF IHC staining and interpreted the results independently, categorizing their responses as positive, negative, or others (with descriptive details). Staining technique details, including antibody clone, dilution, platform (if using an auto-stainer), and IHC assay protocol (In vitro diagnostics (IVD) antibody with IVD protocol, research-use only (RUO) antibody with laboratory-developed test (LDT) protocol, or IVD antibody with LDT protocol), were also submitted. The staining slides were sent back to the Proficiency Testing Committee for a central review by two expert pathologists (Y.-A.C. and J.-F.H.).

**Fig. 1:**
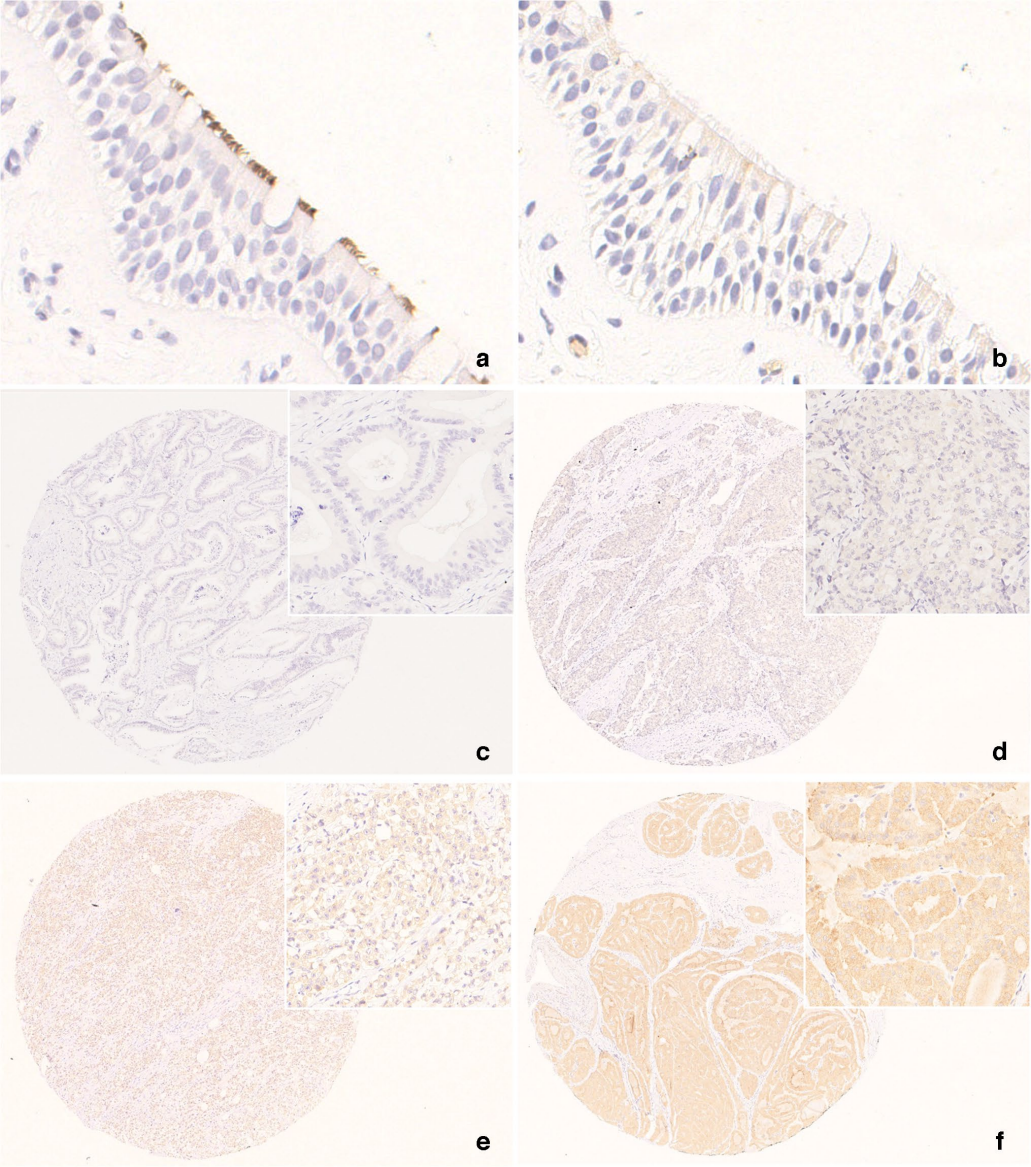
VE1 antibody immunohistochemistry in tissue microarray for proficiency testing. Respiratory cilia should be stained in a strong intensity and served as an external positive control (800x) (**a**), while two participants failed from initial control check because of false negative staining (800x) (**b**). Representative images for 0, 1 +, 2 + and 3 + were demonstrated, respectively (**c-f**)

For the initial control check, any slides showing strong staining on the cilia of the respiratory mucosa was considered passed (Fig. 1a). The concordance and inter-laboratory variability of participant responses were evaluated. The accuracy of participant responses was assessed using molecular testing results as the standard reference (Table 1). Equivocal staining results typically trigger subsequent molecular testing and were therefore considered concordant to the molecular testing result for analysis.

For staining quality assessment,"optimal,""under-staining,"or"over-staining"were categorized based on the following criteria: slides with a false-negative result in core 5 were classified as"under-staining,"while slides with a false-positive result, defined as granular cytoplasmic staining clearly distinguishable from the background, in core 6 were categorized as"over-staining". These results were reviewed and discussed by the two expert reviewers to reach a consensus.

For interpretation quality assessment, two expert pathologists (Y.-A.C. and J.-F.H.) independently reviewed each slide using criteria adapted from Capper et al. [8]. Slides showing unequivocal cytoplasmic staining were graded 1 +, 2 +, or 3 + according to intensity; complete absence of staining was considered 0 (Fig. 1c–f). The 1 + staining was extended to include diffuse faint expression clearly visible under high-power magnification (more than × 40) [13]. Any discrepancies in the intensities were resolved through consensus review of virtual slides. The concordance rate between the reviewers and participant responses were subsequently evaluated.

Statistical analyses were performed using R (version 4.4.2; R Foundation, Vienna, Austria). Fisher’s exact test with Monte Carlo simulation (10,000 replicates) assessed staining quality associations with antibody clone, platform, and assay, while the Kruskal–Wallis test evaluated dilution effects. Inter-laboratory variability was measured by Fleiss'kappa (0–1, with higher values indicating greater agreement). Data visualization was done using"ggplot2"[25].

## Results

A total of 26 pathology laboratories registered for the 2024 BRAF IHC PT, with 15 located in northern Taiwan, five in central Taiwan, and six in southern Taiwan. The most commonly used clone of BRAF antibody was VE1 from Ventana (n = 12, 46.2%) and from Abcam (n = 11, 42.3%), followed by IHC 600 from GenomeMe (n = 3, 11.5%). The dilution folds utilized were as follows: 1:1 (n = 10, 38.5%), 1:2 (n = 2, 7.7%), 1:30 (n = 1, 3.8%), 1:50 (n = 3, 11.5%), 1:100 (n = 7, 26.9%), 1:150 (n = 2, 7.7%), and 1:400 (n = 1, 3.8%). For chromogenic detection, peroxidase with diaminobenzidine (DAB) was used in 24 laboratories (92.3%), and fast red was used in 2 laboratories (7.7%). All tests were performed on autostainers, including Ventana (n = 16, 61.5%), Leica (n = 9, 34.6%), and Dako (n = 1, 3.8%). The IHC assays varied significantly among the institutions, with seven using IVD antibodies with IVD protocols (n = 7, 26.9%), others using IVD antibodies with LDT protocols (n = 5, 19.2%), or RUO antibodies with LDT protocols (n = 14, 53.9%). The details of the methodologies from all institutes are summarized in Table 2.

**Table 2:** Summary of staining methods

Methods	N	%
Antibody clone		
Clone VE1		
Ventana, predilute (IVD), cat no.:760–5095	12	46.2
Abcam, cat no.: ab228461	11	42.3
Clone IHC 600		
GenomeMe, cat. no.: IHC 600–100	3	11.5
Dilution fold		
1:1	10	38.5%
1:2	2	7.7%
1:30	1	3.8%
1:50	3	11.5%
1:100	7	26.9%
1:150	2	7.7%
1:400	1	3.8%
Autostainer platform		
Ventana	16	61.5
BenchMark ULTRA	12	46.2
Benchmark XT	3	11.5
Benchmark GX	1	3.8
Leica	9	34.6
BOND-III	8	30.8
BOND max	1	3.8
Dako Autostainer Link Omnis	1	3.8
IHC assay		
RUO Ab LDT protocol	14	53.8
IVD Ab IVD protocol	7	26.9
IVD Ab LDT protocol	5	19.2
Detecting system		
Fast red	2	7.7%
DAB	24	92.3%

*DAB* diaminobenzidine; *IHC* immunohistochemistry; *IVD* in vitro diagnostics; *LDT* laboratory developed test; *RUO* research use only

All 26 participating laboratories returned their test slides for central expert review. Among them, two laboratories failed from the initial control check due to exhibiting false-negative results in the positive control (Fig. 1b). Notably, both laboratories utilized IVD antibody with LDT protocol on a different manufacturer's platform (Ventana VE1 predilute Ab + Leica BOND-III auto-stainer). The two laboratories were excluded for the substantial analysis.

Among the remaining 24 institutions, there was 100% concordance in the responses of cores 1, 2, 3, and 6, which exhibited staining intensities of either 0 or 3 +. In contrast, participant responses of core 4 failed to reach consensus, with a nearly equal distribution of positive (n = 12, 50%) and negative (n = 10, 41.67%) results, along with two laboratories reporting equivocal findings (8.33%). In core 5, the consensus of most participants (n = 20, 83.33%) was negative, with only 4 (16.67%) participants reported as positive. Overall, the 24 participants demonstrated fair agreement in their responses across all cores, with a Fleiss’ Kappa value of 0.66 (95% CI, 0.6–0.7; *p* < 0.001). The accuracy of participant responses for core 1, 2, 3 and 6 reached 100%. However, the accuracy in core 4 was 58.33%. In core 5, the participant consensus was contrary to the test result, with an accuracy of only 16.7%. The overall accuracy of participant responses was 79.2% (114/144 cores). The sensitivity was 58.3%, while specificity reached 100%. Table 3 demonstrated the summary of participant responses.

**Table 3:** Summary of the participants’ responses

	Core 1	Core 2	Core 3	Core 4	Core 5	Core 6
Positive	0	24	0	12	4	0
Negative	24	0	24	10	20	24
Equivocal	0	0	0	2	0	0
Total	24	24	24	24	24	24
Molecular results	Negative	Positive	Negative	Positive	Positive	Negative
Accuracy	100%	100%	100%	58.33%*	16.67%	100%

*Two labs reported equivocal results and were consider concordant

### Review of staining quality

**Fig. 2:**
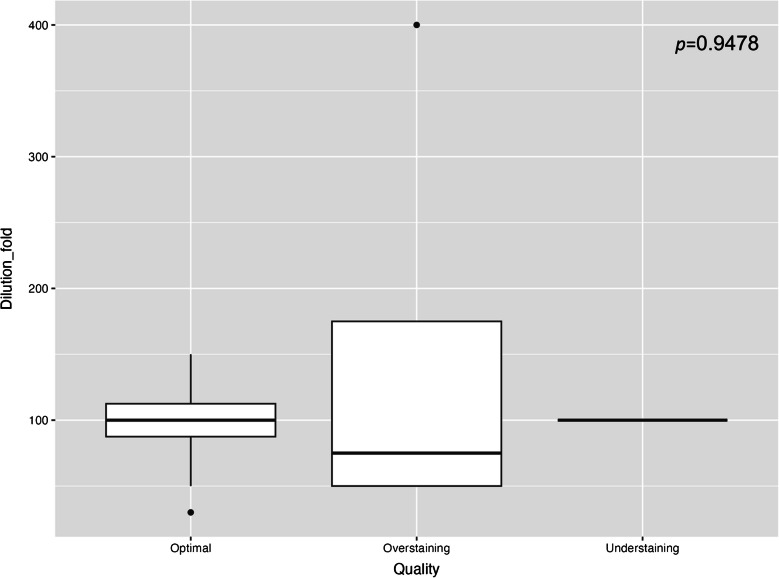
Boxplot demonstrated the relationships between dilution folds and staining quality

**Fig. 3:**
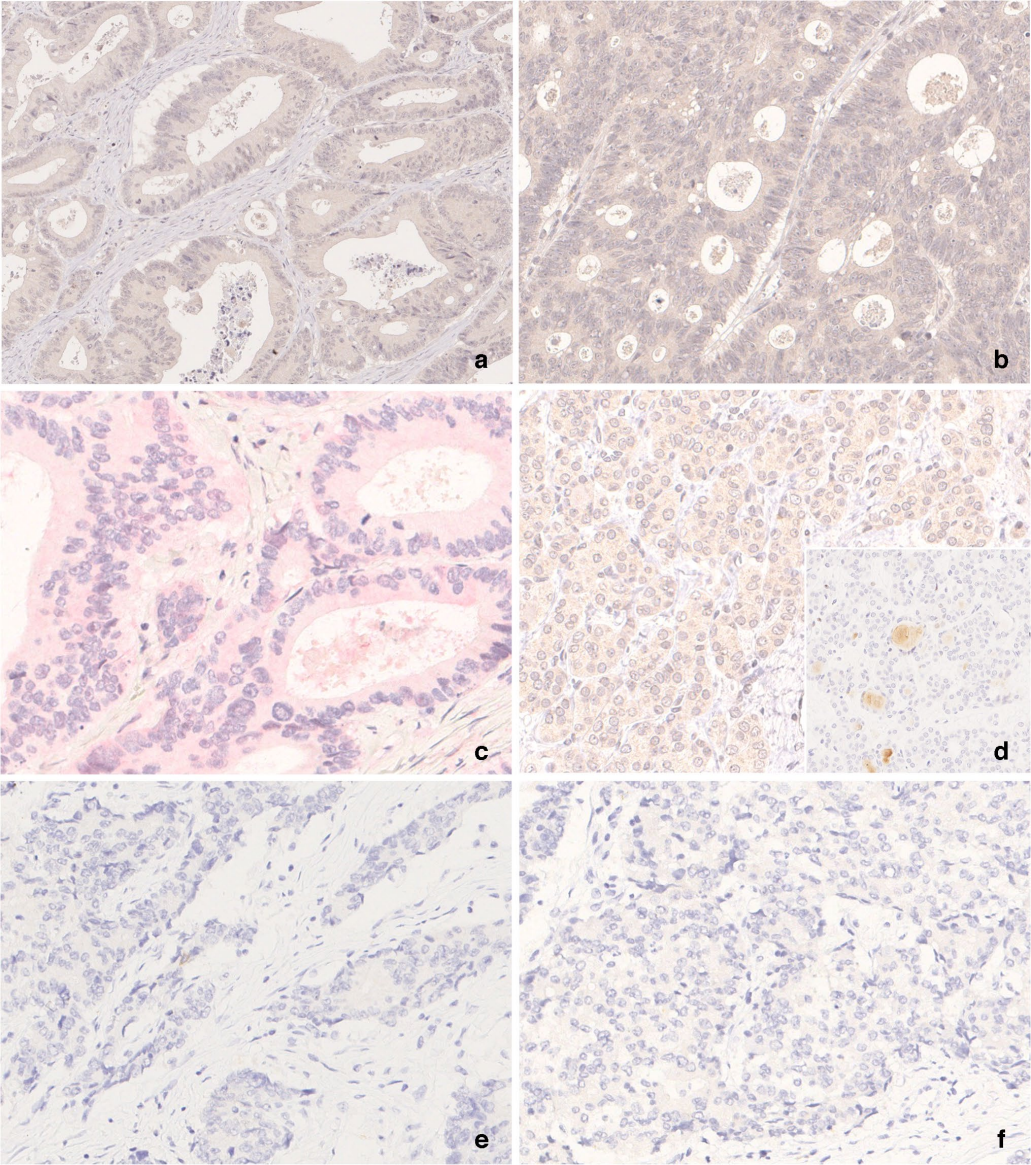
Representative images from over-staining and under-staining samples. (**a**) False positive staining in core 6, colorectal cancer (laboratory 23, 400x). (**b**) False positive staining in core 3, colorectal cancer (laboratory 23, 400x). (**c**) False positive staining using fast-red chromogen, colorectal cancer (laboratory 3, 400x). (**d**) False positive staining in core 1, NIFT-P (laboratory 23, 400x). (**e**–**f**) False negative staining in core 5, colorectal cancer (laboratory 15 and laboratory 16, 400x)

Among the 24 laboratories, 17 (70.83%) achieved optimal results, five (20.83%) exhibited over-staining, and two (8.33%) displayed under-staining. No significant association was found between staining quality and antibody clones (*p* = 0.42), platforms (*p* = 0.08), or IHC assays (*p* = 0.28) (Table 4). Additionally, no significant relationship was observed between antibody dilution and staining quality among 14 laboratories using RUO antibodies with further dilution (Fig. 2, *p* = 0.95). Notably, within the overstaining group, eight cores showed non-specific cytoplasmic staining, including seven with *BRAF* wild-type colorectal tissue. These involved both fast red (2/2) and DAB (3/22) detection systems (Fig. 3a–d). Additional examples of over- and under-staining are shown in Fig. 3e–f.

**Table 4:** Relationships between staining quality and methods

	Optimal	Over-staining	Under-staining	*P*-value
Antibody Clone				0.42
VE1 (Ventana)	9	1	0	
VE1 (Abcam)	6	3	2	
IHC 600 (GenomeMe)	2	1	0	
Platform				0.08
Ventana	13	3	0	
Leica	4	1	2	
Dako	0	1	0	
IHC assay				0.28
RUO Ab LDT protocol	7	4	2	
IVD Ab IVD protocol	7	0	0	
IVD Ab LDT protocol	3	1	0	

*IHC* immunohistochemistry; *IVD* in vitro diagnostics; *LDT* laboratory developed test; *RUO* research use only

### Review of interpretation

**Fig. 4:**
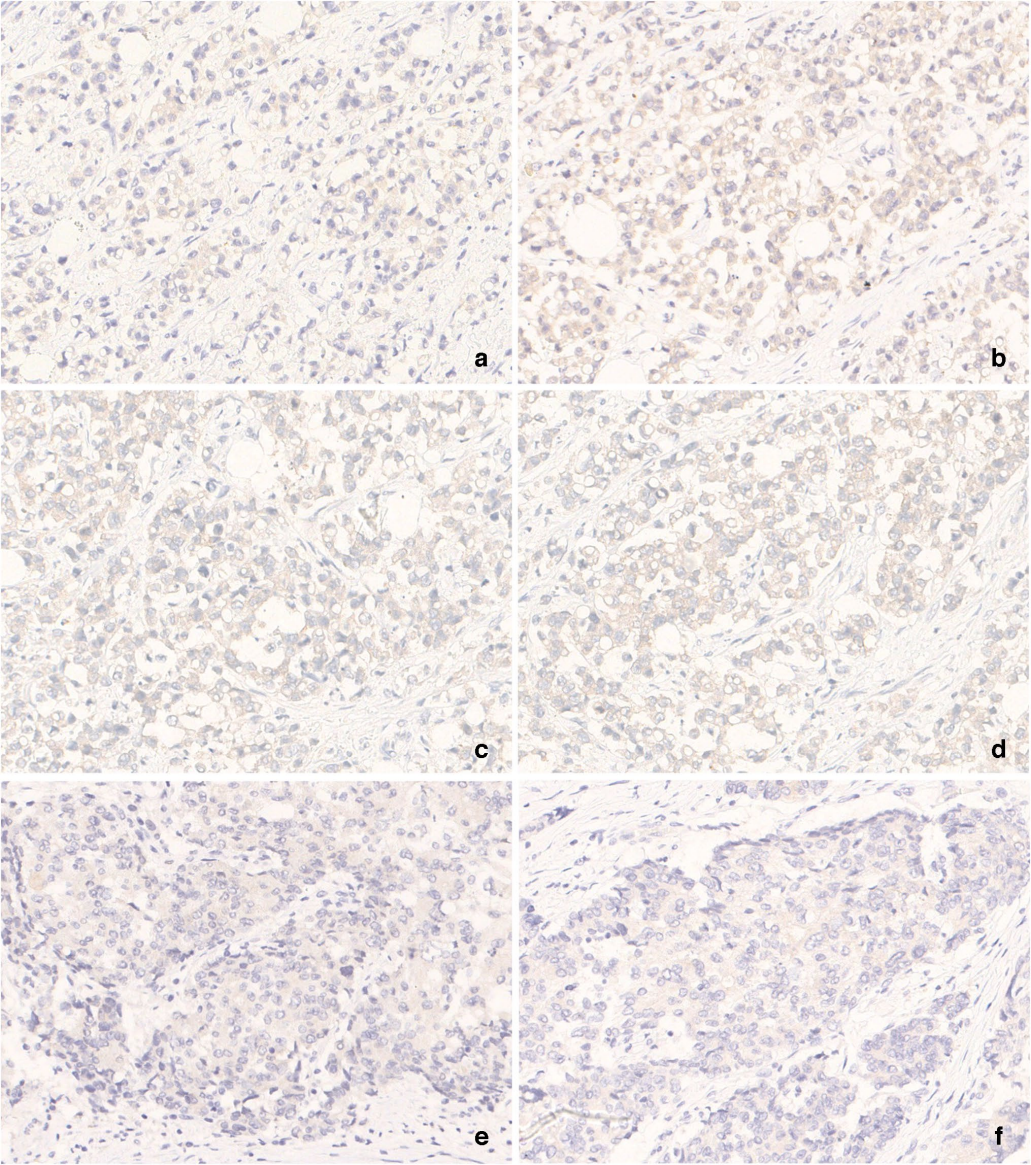
Representative images from cores under-called by participants. (**a**) Core 4, 1 + intensity with participant response as negative (400x). (**b**) Core 4, 2 + intensity with participant response as negative (400x). (**c**) Core 4, 2 + intensity participant response as equivocal (400x). (**d**) Core 4, 2 + intensity with participant response as equivocal (400x). (**e**) Core 5, 1 + intensity with participant response as negative (400x). (**f**) Core 5, 1 + intensity with participant response as negative (400x)

The concordance rate between reviewers and participants varied across institutions, with 100% agreement (6/6 cores) observed in one laboratory (4.17%), 83% agreement (5/6 cores) in twelve laboratories (50%), 67% agreement (4/6 cores) in nine laboratories (37.5%), and 50% agreement (3/6 cores) in two laboratories (8.33%). Overall, the agreement between reviewers and participants was 75% (108 out of 144 cores). All 36 cores with discrepancies between reviewers and participants exhibited issues of under-interpretation of 1 + or 2 + intensity as negative by participants. Specifically, in five laboratories with over-staining issues, eight cores from core 1, 3, and 6 showed false positives (1 + intensity) but were reported as “negative” by their institutions. In core 4, staining ranged from 1 + (n = 7) to 2 + (n = 17) across all slides. Despite this, ten laboratories initially reported them as negative (Fig. 4a-b), while two with 2 + staining reported them as equivocal (Fig. 4c-d). For core 5, 22 slides showed staining intensities of 1 + (n = 18) or 2 + (n = 4). Notably, all slides with a staining intensity of 1 + were reported as negative by the original institutions (Fig. 4e-f). Overall, the most frequent issue was the misinterpretation of 1 + staining as negative in 33 cores across 23 laboratories, and three laboratories under-called 2 + staining as negative.

## Discussion

This proficiency test assessed the quality of BRAF immunohistochemistry (IHC) staining and interpretation in thyroid and colorectal cancers across clinical laboratories in Taiwan. Our findings highlight two key challenges: staining variability and interpretation discrepancies. While 100% consensus was achieved for cores with 0 or 3 + staining, significant inconsistencies arose in cores with 1 + or 2 + staining. Compared to molecular testing, overall accuracy was 79.2%, with 100% specificity but only 58.3% sensitivity. Over-staining (20.83%) was more prevalent in colorectal cancer specimens, while under-staining (8.33%) remained a concern. A 1 + staining control is therefore recommended to help detect under-staining. Interpretation concordance between reviewers and participants was 75%, with all discrepancies due to under-calling of 1 + and rarely 2 + cases. Overall, our results emphasize the need for continuing education focused on technical standardization, the use of appropriate controls, and interpretation criteria to improve diagnostic quality and reduce inter-laboratory variability.

In our study, we assessed staining quality among participants and evaluated the relationship with key analytical parameters, including antibody clone, platform, dilution fold, and IHC assay. Statistical analysis revealed no significant associations between these parameters and staining quality, aligning with most prior reports. Among the examined parameters, antibody clone performance is of particular interest. In our proficiency test, the widely adopted VE1 clone (n = 23) demonstrated robust performance. Notably, three laboratories that using the previously unreported clone IHC600 also achieved acceptable quality, with no statistically significant difference observed between the two clones. These findings suggests that IHC600 may represent a potential alternative to VE1 for clinical application. However, further validation in a larger cohort is warranted to confirm its reliability and diagnostic utility.

While previous studies have found limited correlation between antibody dilution and staining quality, Kuan et al. reported that dilutions above 200-fold may reduce nonspecific nuclear staining [16, 20, 21]. In contrast, the only laboratory in our cohort using a 400-fold dilution, applying the VE1 clone from Abcam on the Dako platform with an LDT protocol, exhibited mild cytoplasmic over-staining. As other detailed information, such as antigen retrieval and detection systems, was not collected, the basis for this finding remains unclear. Regarding staining platforms, earlier studies have generally concluded that platform choice alone does not significantly affect staining outcomes [16, 17], which is consistent with our findings.

In addition, two laboratories in our cohort demonstrated false-negative staining on positive controls and failed the initial quality control; both employed the VE1 antibody from Ventana using the Leica Bond-III platform with an LDT staining protocol. However, a large retrospective study on colorectal cancer reported comparable performance with this combination [16]. Similarly, Estrella et al. found that VE1 staining quality was not affected by the choice of automated stainer (Ventana vs. Leica) [17]. In thyroid cancer studies, only two reports have utilized this combination: one reported 100% sensitivity and 90.63% accuracy [26], while the other reported 88.89% sensitivity and 79.12% accuracy [27]. These discrepancies in reported performance metrics with the same antibody-platform combination indicate that current evidence is insufficient to consider this combination inherently problematic. The observed staining issues may instead reflect laboratory-specific variables not captured in our dataset.

Our study also found that overstaining was more frequently observed in colorectal cancer samples, particularly in the mucin-rich core (core 6), consistent with previous reports [4, 12, 16, 17, 20, 28]. This observation highlights that intrinsic tissue characteristics may contribute to staining variability. Taken together, our findings suggest that the evaluated analytical parameters do not significantly influence staining quality. To improve assay reliability, we recommend that validation protocols incorporate samples from diverse tissue types and that routine practice utilize positive control materials with weak to moderate staining intensity.

**Fig. 5:**
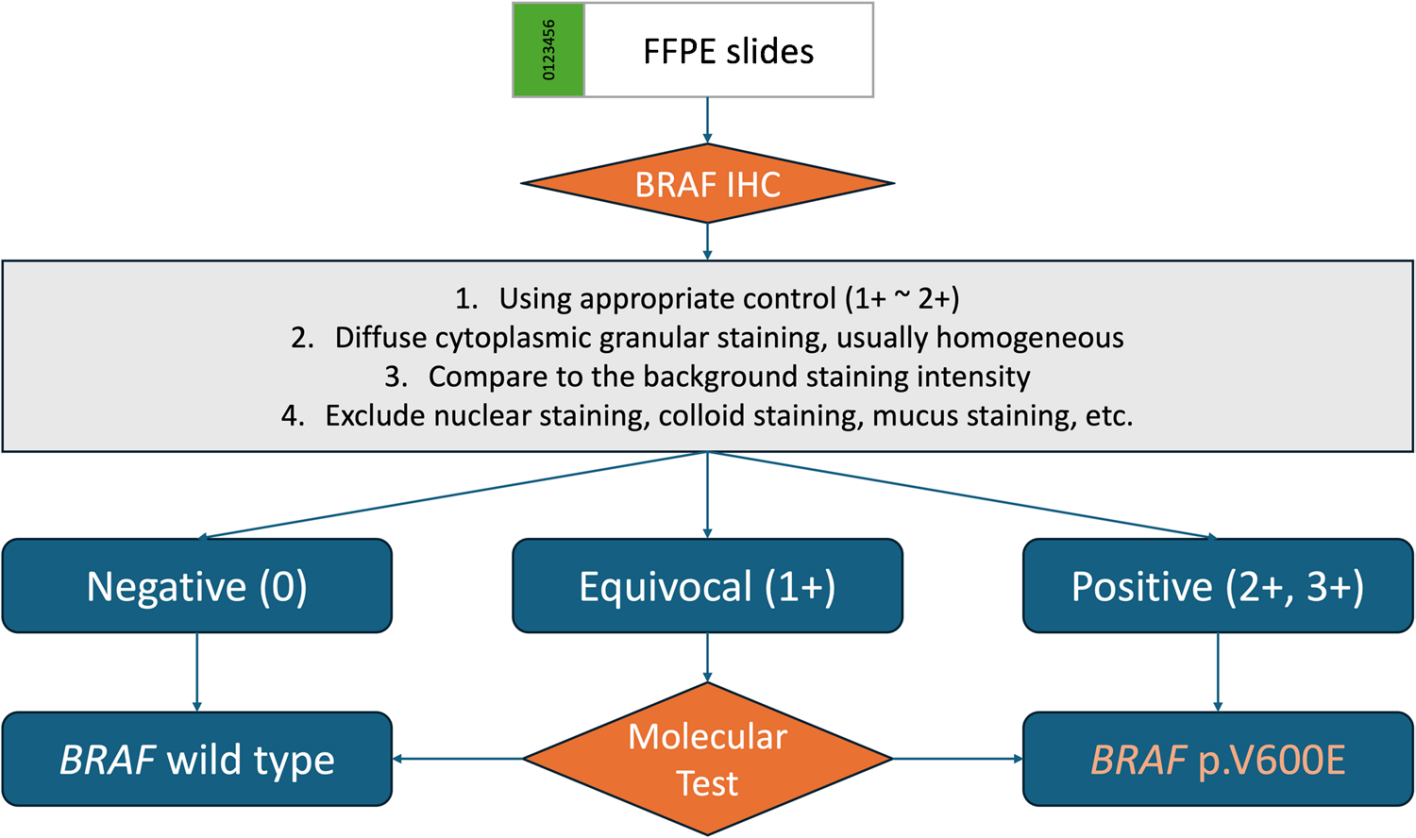
Algorithmic approach integrating BRAF immunohistochemistry with molecular confirmation

Currently, there is no universally accepted standard for IHC interpretation, with most studies utilizing criteria based on the percentage of stained tumor cells and staining intensity, tailored to their specific needs [16, 21]. The most widely adopted standard, described by Capper et al., defines positive cases as those with unambiguous cytoplasmic staining in a substantial portion of viable tumor cells, and faint diffuse staining, isolated nuclear staining, weak staining of scattered cells, or staining in monocytes or macrophages is considered negative [8, 29]. However, weak staining remains a major challenge, affecting diagnostic consistency. For instance, in colorectal cancer, non-specific staining complicates interpretation, with weak staining presenting challenges in approximately 10% of cases [30]. Similarly, in both thyroid and colorectal cancers, 1 + staining is variably classified as negative [16, 21, 31–33] or equivocal, warranting molecular testing [13, 20]. Our findings indicate that most general pathologists interpret weak staining as negative, with varied interpretations for weak-to-moderate staining. Even experienced pathologists struggle with weak staining evaluation. Furthermore, our previous study revealed that 1 + staining can be occasionally observed in *BRAF* wild-type colorectal cancers [13]. Therefore, we recommend generally adhering to the criteria proposed by Capper et al., with an expanded definition of 1 + staining to include diffuse faint cytoplasmic expression, and considered 1 + as equivocal. As supported by several previous studies [13, 20, 30, 34] and illustrated in Fig. 5, molecular testing is recommended for all cases interpreted as 1 + to ensure diagnostic accuracy. Standardizing the interpretation of weak staining may help reduce interobserver variability and improve clinical reliability.

The main limitation of this pilot PT study is its representativeness, involving only 26 participants and six tissue cores. Additionally, focusing solely on thyroid and colorectal neoplasms may limit applicability to other cancers, as BRAF IHC performance varies by tumor origin. Despite these limitations, this PT provides a valuable overview of BRAF IHC performance in Taiwan, identifying key staining and interpretation issues to guide improvements. Future rounds of PT should expand case diversity and include comprehensive documentation of technical parameters, such as incubation times, antigen retrieval methods, and amplification systems, to enable more standardized comparisons and foster inter-laboratory harmonization.

In conclusion, our PT survey evaluated BRAF IHC staining and interpretation in Taiwanese pathology laboratories. We recommend using control samples from diverse tissue sources, including those with staining intensities ranging from 1 + to 2 +, to better calibrate interpretation thresholds. Given the tendency for conservative interpretation of weak staining, targeted educational initiatives and the application of confirmatory molecular testing for equivocal (1 +) cases are essential to minimize the risk of false-negative results.

## Data Availability

The data that support the findings of this study are available from the corresponding author upon reasonable request.
